# Synovial pH, lactate, and glucose as adjunct biomarkers to exclude periprosthetic joint infection in hip and knee arthroplasty: a prospective study

**DOI:** 10.5194/jbji-10-293-2025

**Published:** 2025-08-13

**Authors:** Dirk Müller, Joshua Aulenbacher, Niels Heine, Benjamin Schloßmacher, Vincent Lallinger, Georg Matziolis, Rüdiger von Eisenhart-Rothe, Igor Lazic

**Affiliations:** 1 Department of Orthopaedic Surgery, School of Medicine, Klinikum rechts der Isar, Technical University of Munich, Ismaninger Str. 22, 81675 Munich, Germany; 2 Orthopaedic Department, Friedrich-Schiller-University Jena, University Hospital Jena, Campus Eisenberg, Klosterlausnitzer Str. 81, 07607 Eisenberg, Germany

## Abstract

**Background**: Synovial pH, glucose, and lactate have been studied in native joint septic arthritis, but studies in periprosthetic joint infection (PJI) are limited. The purpose of this study is to evaluate the diagnostic utility of synovial pH, lactate, and glucose as potential diagnostic biomarkers for PJI. **Materials and methods**: Joint aspirations were performed in 100 consecutive patients (24 hips, 76 knees) with suspected PJI. In our cohort, 36 % were diagnosed with PJI, while 64 % were classified as uninfected based on the EBJIS criteria of 2021. Receiver operating characteristic (ROC) curves and Youden's index were employed to determine the optimal thresholds for synovial pH, lactate, and glucose. **Results**: The mean synovial pH was significantly lower in the PJI group compared to the uninfected group (7.23 vs. 7.49; p=0.009 ). The mean synovial lactate was significantly higher in the PJI group compared to the uninfected group (8.81 vs. 6.28; p=0.008 ). The mean synovial glucose was significantly lower in the PJI group compared to the uninfected group (41.4 vs. 62.1; p=0.027 ). The optimal thresholds for synovial pH, lactate, and glucose were 7.25, 8.45 mmol L^−1^ and 18 mg dL^−1^ with a sensitivity of 50 %, 50 %, and 30 % as well as a specificity of 81 %, 84 %, and 96 %, respectively. The combined use of all three biomarkers yielded a negative predictive value of 84 % for the diagnosis of PJI. **Conclusion**: Synovial pH, lactate, and glucose may serve as valuable adjunct parameters in the diagnosis of PJI with a high negative predictive value.

## 1 Introduction

Periprosthetic joint infection (PJI) is one of the most concerning complications following total joint arthroplasty with an incidence rate of 1 %–2 % (Kamath et al., 2015; Nikolaus et al., 2016). As the number of joint replacements continues to grow, the frequency of revision surgeries due to PJI is expected to increase correspondingly in the coming years, necessitating an accurate and timely diagnosis of PJI to guide appropriate treatment strategies (Inacio et al., 2017; Zimmerli et al., 2004). Synovial fluid analysis has emerged as a cornerstone in diagnostic algorithms for PJI and plays a critical role in the definitions proposed by the Musculoskeletal Infection Society (MSIS) in 2018 and the European Bone and Joint Infection Society (EBJIS) in 2021 (Parvizi et al., 2018; McNally et al., 2021). Synovial leukocyte count and the percentage of polymorphonuclear neutrophils (PMN %) have long been considered essential biomarkers in diagnostic algorithms (Sabater-Martos et al., 2024; Zmistowski et al., 2012). In addition to these conventional parameters, several other synovial biomarkers have been investigated to enhance the diagnostic accuracy for PJI. Synovial C-reactive protein (CRP), interleukin levels, and interferon- γ have all shown potential diagnostic utility in differentiating PJI from aseptic arthroplasty failures (Lee et al., 2017). Recent research has focused on novel synovial biomarkers such as alpha-defensin, calprotectin, and D-lactate, which have demonstrated promising diagnostic potential (Lazic et al., 2022; Li et al., 2021; Renz et al., 2018). However, an optimal universal diagnostic biomarker has yet to be identified (Shahi and Parvizi, 2016).

Synovial pH level, glucose, and lactate levels are well-known parameters in diagnosing native joint septic arthritis (Ravn et al., 2023; Shirtliff Mark and Mader Jon, 2002; Gobelet and Gerster, 1984; Lenski and Scherer, 2014a; Carpenter et al., 2011; Riley, 1981; Brennan and Hsu, 2012; Elsissy et al., 2020; Bo Söderquist, 1998). Interestingly, these widely available biomarkers, which can be rapidly and affordably assessed, have not been extensively studied in the context of PJI. Only a limited number of studies have investigated the diagnostic utility of pH levels, lactate, and glucose in arthroplasties, yielding heterogeneous results and even contradictory thresholds (Theil et al., 2022; Judl et al., 2024; Sabater-Martos et al., 2025b). The purpose of the study is to analyze the distinct and combined diagnostic utility of synovial pH, lactate, and glucose as potential biomarkers for PJI.

## 2 Materials and methods

In this prospective study, we included patients scheduled to undergo revision surgery for chronic septic or aseptic failure of hip or knee arthroplasties. All patients with chronic systemic inflammation, recent surgery in the past 4 weeks, malignancies, or a concurrent acute infection in any other organ system were excluded from the study. A total of 100 patients met the study's inclusion criteria and were prospectively evaluated (24 hips, 76 knees). Patients were classified as aseptic (infection unlikely) or PJI (infection confirmed) according to the EBJIS criteria of 2021 (McNally et al., 2021). Patients in the category “infection likely” ( n=3 ) were included in the PJI group. Joint aspirations were performed under sterile conditions in our outpatient department after an antibiotic-free interval of at least 2 weeks, when necessary. Hip joint aspirations were performed under fluoroscopic guidance. Synovial pH levels were immediately measured with a calibrated pH analyzer (Seven Easy, Mettler-Toledo, Columbus, Ohio, USA) at room temperature (23 °C). Synovial fluid lactate and glucose were analyzed using a clinical chemistry analyzer (Cobas^®^ c702, Roche Diagnostics, Basel, Switzerland). Synovial white blood cell count (WBC) and polymorphonuclear percentage (PMN %) were analyzed with an automatic Sysmex XN-2000 hematology analyzer (Sysmex Corporation, Kobe, Japan) in body fluid mode. Furthermore, synovial fluid was cultured for 14 d in both aerobic and anaerobic blood culture bottles using the BACTEC^TM^-system (BD, Sparks, MD, USA). If microbiological growth was detected without pathogen identification, the corresponding samples were subsequently cultured in thioglycolate broth to facilitate further microbial detection.

During revision arthroplasty, five intraoperative tissue samples were collected for microbiology cultures, and one tissue sample was taken for histological analysis. The periprosthetic membrane was classified according to Morawietz and Krenn (Krenn et al., 2013). Ethical approval was waived by the local Ethics Committee of the Technical University Munich (IRB approval no. 714/20S, 20 November 2020). Informed consent was obtained from all individual participants included in the study.

### Statistical analysis

Data collection and statistical analysis were performed using Excel (Microsoft Corporation, Redmond, WA, USA) and the Statistical Package for the Social Sciences Statistics for Windows version 29 (IBM Corporation, Armonk, NY, USA). The Shapiro–Wilk test was used to assess whether the variables followed a normal distribution. Measures that were tested to conform to a normal distribution were expressed as mean ± standard deviation (SD), and those that did not conform to a normal distribution were expressed as median (quartile 1 – quartile 3, $Q_{1}$ – $Q_{3}$ ). According to the normality test, independent two-sample t tests or Mann–Whitney U tests were used for continuous variables. Frequencies were calculated for categorical variables, and comparisons were made using the $\chi^{2}$ test. Statistical evaluation was performed with receiver operating characteristic (ROC) analyses presenting the area under the curve (AUC) with a 95 % confidence interval (CI). The optimal cutoff values for synovial pH levels, lactate, and glucose were determined using Youden's index. Differences were considered statistically significant if the p value was ≤0.05 .

## 3 Results

PJI was identified in 36/100 cases (10 hips, 26 knees). In 29 of 36 cases (80.6 %) of PJI, a causative pathogen was identified (Table 1), while 7 (19.4 %) patients had culture-negative infections and 4 (11.1 %) had a polymicrobial infection.

**Table 1:** List of bacteria identified in the 36 patients from the PJI group.

Culture organism	Frequency
	(%)
Culture-negative	19.4 %
*Staphylococcus epidermidis*	19.4 %
*Staphylococcus aureus*	11.1 %
*Staphylococcus lugdunensis*	5.6 %
*Staphylococcus petrasii*	2.8 %
*Enterococcus faecalis*	8.3 %
*Enterococcus gallinarum*	2.8 %
*Cutibacterium acnes*	2.8 %
*Cutibacterium granulosum*	2.8 %
*Cutibacterium avidum*	2.8 %
*Streptococcus dysgalactiae*	5.6 %
*Streptococcus mutans*	2.8 %
*Klebsiella oxytoca*	2.8 %
*Escherichia coli*	2.8 %
*Pseudomonas oleovorans*	2.8 %

Patient demographics are presented in Table 2. No significant differences were observed between the PJI and aseptic groups in age, gender distribution, number of previous surgeries, BMI, ASA classification, or prevalence of diabetes.

**Table 2:** Patient characteristics.

Characteristics	Not infected	PJI	p value
	( n=64 )	( n=36 )	
Age (years) (mean, SD)	70.7 (13.2)	67.2 (10.8)	0.173
Gender (female/male, %)	62/38	56/44	0.496
Laterality (left/right, %)	55/45	50/50	0.652
Number previous surgeries (median, $Q_{1}$ – $Q_{3}$ )	2 (1–4)	2 (1–5)	0.293
BMI (median, $Q_{1}$ – $Q_{3}$ ) (kg m^−2^)	28.3 (26.0–34.0)	29.9 (25.9–37.0)	0.586
ASA II–II/III–IV (%)	60.0/40.0	48.6/51.4	0.279
Diabetes (%)	17.2	13.9	0.666

### 3.1 Synovial pH level

**Figure 1:**
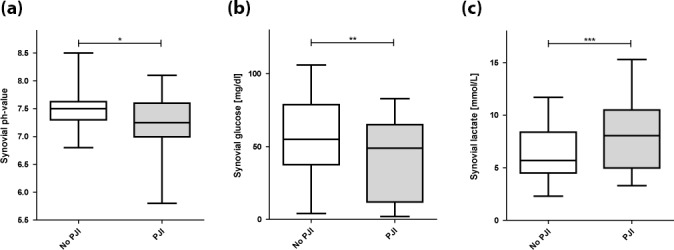
Synovial pH, glucose, and lactate in periprosthetic joint infection (PJI) and uninfected joints (^*^ p=0.009 ; ^**^ p=0.027 ; ^***^ p=0.008 ).

**Figure 2:**
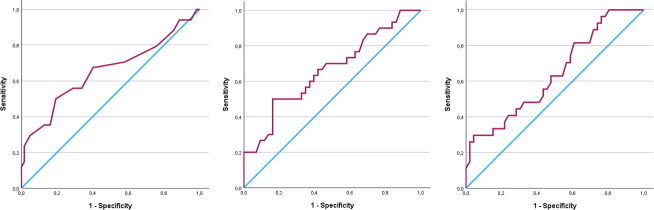
Receiver operating characteristics curves for synovial pH (AUC = 0.654), lactate (AUC = 0.662), and glucose (AUC = 0.640).

The mean synovial pH level was significantly lower in patients diagnosed with PJI (7.23 ( ±0.51 )) compared to the uninfected group (7.49 ( ±0.30 )) ( p=0.009 ) (Table 3, Fig. 1). The ROC curve analysis for pH showed an AUC of 0.654 (95 % CI, 0.531–0.777) (Fig. 2). The optimal threshold for synovial pH was determined to be 7.25 with a sensitivity of 50 % and specificity of 81 % (Table 4).

**Table 3:** Results for synovial pH, lactate, and glucose in PJI and uninfected arthroplasties.

Markers	Not infected	PJI	p value
	( n=64 )	( n=36 )	
Synovial pH (mean, SD) ( n=96 )	7.49 (0.30)	7.23 (0.51)	0.009
Synovial lactate (mean, SD) ( n=73 ) (mmol L^−1^)	6.28 (2.55)	8.81 (5.06)	0.008
Synovial glucose (mean, SD) ( n=73 ) (mg dL^−1^)	62.1 (42.9)	41.4 (26.9)	0.027

**Table 4:** Cutoff values for synovial pH, lactate, and glucose. Optimal cutoff values were determined by analyzing the ROC curves and calculating Youden's Index.

Markers	Used cutoff	AUC	Sensitivity	Specificity	PPV	NPV	Accuracy
	values						
Synovial pH ( n=96 )	7.25	0.654	50 %	81 %	59 %	75 %	70 %
Synovial lactate ( n=73 )	8.45 mmol L^−1^	0.662	50 %	84 %	68 %	71 %	70 %
Synovial glucose ( n=73 )	18 mg dL^−1^	0.640	30 %	96 %	80 %	70 %	71 %
Combined ( n=68 )	combined (or)	–	81 %	62 %	57 %	84 %	69 %

### 3.2 Synovial lactate

The mean synovial lactate level was significantly higher in the patients diagnosed with PJI (8.81 mmol L^−1^ ( ±5.06 )) compared to the uninfected group (6.28 mmol L^−1^ ( ±2.55 )) ( p=0.008 ) (Table 3, Fig. 1). The ROC curve analysis for lactate showed an AUC of 0.662 (95 % CI, 0.534–0.790). The optimal threshold for synovial lactate was determined to be 8.45 mmol L^−1^ with a sensitivity of 50 % and specificity of 84 % (Table 4).

### 3.3 Synovial glucose

The mean synovial glucose level was significantly lower in the patients diagnosed with PJI (41.4 mg dL^−1^ ( ±26.9 )) compared to the uninfected group (62.1 mg dL^−1^ ( ±42.9 )) ( p=0.027 ) (Table 3, Fig. 1). The ROC curve analysis for glucose showed an AUC of 0.640 (95 % CI, 0.509–0.771). The optimal threshold for synovial glucose was determined to be 18.0 mg dL^−1^ with a sensitivity of 30 % and specificity of 96 % (Table 4). In diabetic patients ( N=16 ), the mean synovial fluid glucose level was higher (93.7 mg dL^−1^ ( ±72.5 )) than in non-diabetic patients (48.2 mg dL^−1^ ( ±26.6 )) ( n=84 ) without reaching statistical significance ( p=0.08 ).

### 3.4 Combination of synovial pH, lactate, and glucose

In a combined assessment of all three biomarkers (synovial fluid pH ≤ 7.25 or lactate ≥ 8.45 mmol L^−1^ or glucose ≤ 18 mg dL^−1^), the shared diagnostic utility yielded a sensitivity of 81 %, a specificity of 62 %, a positive predictive value (PPV) of 57 %, and a negative predictive value (NPV) of 84 % (Table 4).

## 4 Discussion

The principal finding of this study is that all three investigated synovial biomarkers can significantly differentiate between PJI and aseptic cases in this cohort. Moreover, the combined analysis of synovial pH, lactate, and glucose levels demonstrates a high NPV of 84 %. This suggests that these parameters, when used together, offer a practical and prompt diagnostic tool to likely exclude PJI without the need to rely on more complex diagnostic tests.

### 4.1 Synovial pH level

The literature on pH levels as a diagnostic biomarker for PJI is scarce. It has only recently been demonstrated to have favorable diagnostic utility (Theil et al., 2022; Judl et al., 2024). Despite variations in reported cutoff values, previous research consistently demonstrates that synovial pH is significantly lower in PJI compared to uninfected arthroplasties. These variations likely reflect differences in measurement techniques, underscoring the need for standardized measurement protocols. Theil et al. (2022) investigated synovial pH levels using a commercial blood gas analyzer. Their reported threshold was significantly more acidic than our findings (7.11 vs. 7.25), indicating that discrepancies between studies may stem from variations in calibration protocols of different instrumentation. Although both results were satisfactory, it is important to acknowledge that commercial blood gas analyzers are not specifically calibrated to analyze synovial fluid. Conversely, Judl et al. (2024) reported a higher pH threshold compared to our findings (7.4 vs. 7.25). Notably, their study observed a marked divergence in mean pH levels between the infected and uninfected cohorts, with the mean pH of the uninfected prostheses being considerably more alkaline at 7.8. Although their methodology appears broadly comparable to ours, their study lacks a comprehensive description of the statistical methods used to determine their threshold value, which raises concerns regarding the direct comparability of these findings. These discrepancies underscore the need for standardized protocols in the measurement of synovial pH to ensure the validity and reproducibility of results across different studies. In this regard, it is important to consider the fact that pH is a highly sensitive parameter influenced by a variety of physiological and pathological factors, including electrolyte imbalances, inflammatory responses, systemic infections, and temperature variations (Ashwood et al., 1983).

Interestingly, the mean pH of uninfected cases in our study (7.49) closely aligns with reported physiological pH values of native joints, which typically range between 7.39 and 7.43 (Ropes and Bauer, 1953; Cummings and Nordby, 1966). Milošev et al. (2017) hypothesized that metal ion release from arthroplasties may contribute to a reduction in synovial pH, distinguishing it from the pH observed in native joints. Consequently, it is plausible that synovial pH could be influenced by factors such as implant size or material composition. Larger implants may result in a more pronounced decrease in synovial pH and, therefore, lead to false positive PJI cases. In our cohort, the pH levels of uninfected arthroplasties were predominantly neutral with a tendency toward alkalinity. Thus, the influence of joint replacement on synovial fluid pH remains open and should be addressed in future studies.

### 4.2 Synovial lactate

Synovial fluid lactate has not been well studied as a biomarker in PJI. While lactate would have been the more obvious focus of research, as it is a reliable biomarker in native joint septic arthritis, recent studies have shifted attention to D-lactate. D-lactate is almost exclusively produced by bacteria and has demonstrated promising diagnostic performance in PJI with pooled sensitivity and specificity of 82 % and 76 %, respectively (Li et al., 2021). Some studies have also reported promising results for the diagnostic utility of lactate in PJI. Sharma et al. (2020) evaluated lactate as a biomarker for PJI, reporting a sensitivity of 69 % and a specificity of 77 % using a threshold of 6.95 mmol L^−1^. Similarly, Lenski and Scherer (2014b, 2015) demonstrated a sensitivity of 73 % and a specificity of 67 % when a threshold of 5.3 mmol L^−1^ was used (Lenski and Scherer, 2015) and a sensitivity of 71 % and a specificity of 88 % when a threshold of 8.3 mmol L^−1^ was used (Lenski and Scherer, 2014b). Our findings are consistent with previously published literature; however, it is noteworthy that the threshold value identified in our study (8.45 mmol L^−1^) lies at the higher end of the range reported in existing studies. Synovial lactate remains a promising and cost-effective biomarker, as supported by this study. Larger studies are needed to exactly describe threshold values and confirm its diagnostic accuracy.

### 4.3 Synovial glucose

Low synovial glucose levels have historically been considered indicative of septic arthritis in native joints (Bo Söderquist, 1998). However, the diagnostic utility of synovial glucose has been questioned due to significant variability in results, a concern previously discussed in the context of native joint infections. In the context of PJI, the available literature is limited. Previous studies reported heterogeneous diagnostic performance of synovial glucose in diagnosing PJI with sensitivities ranging from 56 % to 79 % and specificities between 65 % and 90 % (Lenski and Scherer, 2014b, 2015; De Vecchi et al., 2016; Haertlé et al., 2022; Sabater-Martos et al., 2025b). In particular, the application of glucose strip tests resulted in numerous false positives and false negatives, which are partly attributable to the semi-quantitative output of such test strips (Haertlé et al., 2022). Haertlé et al. (2022) even concluded that the combined semi-quantitative analysis of leukocyte esterase and glucose using test strips demonstrates lower diagnostic accuracy compared to the use of leukocyte esterase alone. Lenski et al. analyzed synovial glucose levels processed in a clinical routine laboratory to diagnose PJI. In a 2014 study including 24 synovial fluid samples, they reported both sensitivity and specificity of 79 % (Lenski and Scherer, 2014b). However, in a subsequent 2015 study involving a larger cohort of 76 samples, the reported sensitivity dropped to 56 % and specificity to 65 % (Lenski and Scherer, 2015). Based on these findings, the authors concluded that synovial glucose alone demonstrates limited diagnostic accuracy for PJI. A recent study by Sabater-Martos et al. (2025b) investigated the utility of synovial glucose for diagnosing acute PJI in a cohort study of 78 patients, reporting a sensitivity of 75 % and a specificity of 90 %. Our findings align with these previous reports; however, our results demonstrated high specificity, placing them within the upper range reported in the existing literature. Notably, our threshold value for synovial glucose (18 mg dL^−1^) was substantially lower than the thresholds reported by Lenski and Scherer (2014b, 2015) (44 and 50 mg dL^−1^ in the 2014 and 2015 studies, respectively) (Lenski and Scherer, 2014b, 2015) and by Sabater-Martos et al. (2025b) (44 mg dL^−1^). This discrepancy could be attributed to differences in study populations. While our study focused exclusively on chronic PJI of the hip and knee, Sabater-Martos et al. (2025b) investigated acute PJI of the knee, and Lenski and Scherer (2014b, 2015) included both acute and chronic PJI across various joints.

One important factor affecting the diagnostic accuracy of synovial glucose is the influence of systemic glucose levels. Previous studies have suggested that fluctuating serum glucose levels may alter synovial glucose concentrations (Brennan and Hsu, 2012), which could also explain the heterogeneity of the reported results in arthroplasties. In our cohort, although this difference did not reach statistical significance, a clear trend toward higher synovial glucose levels was observed in diabetic patients. This observation highlights the need to interpret synovial glucose values in relation to blood glucose levels. Sabater-Martos et al. (2025b) calculated a serum-to-synovial-glucose ratio and determined the optimal cutoff value to be >0.69 for the diagnosis of acute PJI with a sensitivity of 72 % and a specificity of 94 %. Further research is required to establish whether this cutoff also applies to chronic PJI.

### 4.4 Combination of synovial pH, lactate, and glucose

To the best of our knowledge, this is the first study reporting on the combined diagnostic utility of synovial pH, lactate, and glucose levels for PJI diagnostics. A significant clinical advantage of these three biomarkers is that they can be easily and rapidly determined in a routine clinical laboratory setting alongside synovial WBC, and PMN %, which are crucial and mandatory cornerstones of PJI diagnostics. This highlights the clinical potential of the combined assessment of these biomarkers, particularly given the high reported NPV. In this context, it is noteworthy that more complex diagnostic modalities, including leukocyte scintigraphy, PET–CT, and molecular techniques, demonstrate comparable specificities (Jun and Jianghua, 2018; Xing et al., 2013; Kwee et al., 2008). Despite the severity of PJI as a complication, its overall incidence remains low. Consequently, the ability to differentiate inconclusive, presumably aseptic cases using simple, inexpensive tests as part of the existing synovial analysis may represent a useful diagnostic improvement. Furthermore, the combined evaluation of these three simple synovial biomarkers demonstrated a sensitivity approaching that of established diagnostic markers such as synovial WBC, PMN %, and microbiological cultures of sonication fluid (Trampuz et al., 2007; De Fine et al., 2018). Trampuz et al. (2007) reported a sensitivity of 78.5 % for sonication fluid cultures in a study of 331 patients, including 79 with PJI. Moreover, De Fine et al. (2018) reported pooled sensitivities of 89.5 % for synovial WBC count and 88.3 % for PMN % in a meta analysis on the role of synovial fluid analysis for PJI detection which included 2390 patients. While these established biomarkers exhibit markedly higher specificities, granting them superior overall diagnostic accuracy, the findings of this study underscore the potential value of incorporating the simple assessment of these three biomarkers into the already prevailing synovial analysis for PJI diagnostics. Further investigation is also warranted to explore the combined sensitivity of these three biomarkers, especially regarding the exact definition of thresholds.

In a recent meta analysis of 74 studies, Sabater-Martos et al. (2025a) identified rule-in thresholds (specificity > 95 %) of a WBC count ≥ 3000 cells per microliter and PMN ≥ 75 % and rule-out thresholds (sensitivity > 95 %) of a WBC count ≤ 1500 cells per microliter and PMN ≤ 65 %. For future research, similar rule-in and rule-out thresholds could be established for synovial pH, lactate, and glucose.

### 4.5 Limitations

This study has several limitations. First, we exclusively applied the 2021 EBJIS definition (McNally et al., 2021) as the gold standard, which is currently considered the most sensitive among the major definitions (Sousa et al., 2023). Previous classifications, such as the ICM 2018 definition (Parvizi et al., 2018), could potentially yield different results. Thus, this choice limits comparability with studies that have employed alternative gold standards in their analyses. Second, although this is the largest study on synovial pH, lactate, and glucose for the diagnosis of PJI to date, the combined diagnostic utility of these three biomarkers requires confirmation in larger cohorts applying the same measurement techniques and protocols. Third, other leukocyte-derived markers, such as alpha defensin, calprotectin, and leukocyte esterase, should also be included to enable a direct comparison of their diagnostic utility.

### 4.6 Conclusion

In conclusion, the combined assessment of synovial pH, lactate, and glucose levels provides a reliable diagnostic tool for PJI with a high NPV of 84 %. This highlights the clinical utility of these biomarkers as a rapid and cost-effective addition to routine synovial analysis, potentially reducing reliance on more complex and resource-intensive diagnostic methods. Further studies with standardized measurement protocols and larger cohorts are necessary to validate these findings and refine diagnostic thresholds for broader clinical implementation.

## Data Availability

The datasets used and/or analyzed during the current study are available from the corresponding author on reasonable request.

## Appendix A Abbreviations

**Table:**

AUC	Area under the curve
ASA	American Society of Anesthesiologists
BMI	Body mass index
CRP	C-reactive protein
ROC	Receiver operating characteristic
NPV	Negative predictive value
PJI	Periprosthetic joint infection
PMN %	Polymorphonuclear percentage
PPV	Positive predictive value
WBC	White blood cell count
